# Characterization and Anti-Ultraviolet Radiation Activity of Proanthocyanidin-Rich Extracts from *Cinnamomum camphora* by Ultrasonic-Assisted Method

**DOI:** 10.3390/molecules29040796

**Published:** 2024-02-08

**Authors:** Zaizhi Liu, Haibin Liao, Yanting Dai, Yanlong Qi, Zhengrong Zou

**Affiliations:** 1 College of Life Sciences, Jiangxi Normal University, Nanchang 330022, China; hbliao112@163.com (H.L.); daiyanting1119@163.com (Y.D.); 2 Key Laboratory of High–Performance Synthetic Rubber and Its Composite Materials, Changchun Institute of Applied Chemistry, Chinese Academy of Sciences, Renmin Road, Changchun 130022, China; ylqi@ciac.ac.cn

**Keywords:** proanthocyanidin-rich extracts, ultrasonic-assisted extraction, characterization, anti-ultraviolet radiation activity

## Abstract

The ultrasonic-assisted extraction (UAE) method was employed to separate *Cinnamomum camphora* proanthocyanidin-rich extracts (PCEs). This extraction process was optimized by the Box–Behnken design, and the optimal conditions, on a laboratory scale, were as follows: an ethanol concentration of 75%, a liquid-to-solid ratio of 24 mL/g, an ultrasonic time of 39 min, and an ultrasonic power of 540 W. Under the obtained conditions, the PCE yield extracted by UAE was higher than that from heat reflux extraction and soaking extraction. An ultra-performance liquid chromatography–tandem mass spectrometry analysis was employed to characterize the phloroglucinolysis products of the *C. camphora* PCEs, by which epigallocatechin, catechin, epicatechin, and (−)-epigallocatechin-3-*O*-gallate were identified as the terminal units; epigallocatechin, epicatechin, and (−)-epigallocatechin-3-*O*-gallate were recognized as extension units. The *C. camphora* PCEs possessed higher anti-ultraviolet activity in vitro compared with the commercially available sunscreen additive of benzophenone with respect to their ethanol solutions (sun protection factor of 27.01 ± 0.68 versus 1.96 ± 0.07 at a concentration of 0.09 mg/mL) and sunscreens (sun protection factor of 17.36 ± 0.62 versus 14.55 ± 0.47 at a concentration of 20%). These results demonstrate that *C. camphora* PCEs possess an excellent ultraviolet-protection ability and are promising green sunscreen additives that can replace commercial additives.

## 1. Introduction

The ozone layer protects living beings on earth by blocking most of solar radiation [1]. Among the unblocked solar radiation, ultraviolet light has harmful effects on humans. Ultraviolet light consists of ultraviolet-A light (UVA), ultraviolet-B light (UVB), and ultraviolet-C light (UVC) [2,3]. UVA and UVB can pass through the atmosphere, thus causing skin damage. UVC could be intercepted by the ozone layer [2]. Previous studies indicated that the effects of UVA on the skin are not as powerful as UVB, thanks to its cumulative effect [4,5]. Meanwhile, UVB is more absorbed by the epidermis as it has a shorter wavelength, and, hence, it is the more harmful to the skin [6]. Recently, several reports have shown that overexposure to UVB can lead to cellular aging, photoaging, and skin cancers (e.g., non-melanoma and melanoma) [6,7,8]. Furthermore, Narayanan et al. reported that the prevalence of skin cancer is equal to the prevalence of all other organ cancers put together [9].

Sunscreens are a class of lotion-like substances that can prevent sunburn or tanning by chemically absorbing or physically reflecting some ultraviolet radiation [10]. Sunscreens can be divided into organic and inorganic sunscreens based on their basic composition, in which inorganic compounds or synthetic photo-protectants are generally used [11,12]. TiO_2_ and ZnO are the main components in typical inorganic sunscreens, which can lead to skin lesions, oxidative DNA damage, and even cancer [13,14]. Organic sunscreens have greater sun protection than inorganic sunscreens. However, organic sunscreens could cause skin allergies or photo-induced toxicities [15]. Therefore, it is of great significance to find a safer and less-negative sunscreen. Natural sun-protection biological extracts are ideal substitutes for traditional sunscreen ingredients [5,14].

Recently, polyphenol substances (e.g., phenolic acids, flavonoids, and proanthocyanidins) were considered as potential natural skin protectants, owing to their anti-ultraviolet activity, antioxidant capacity, and DNA repair function [16]. *Cinnamomum camphora* is a subtropical evergreen tree, which is extensively cultivated in south China as a source of traditional Chinese herbs [17,18]. *C. camphora* leaves possess various natural active substances, such as alkaloids, terpenoids, phenols, and steroids [19]. Proanthocyanidins, as flavan-3-ol oligomers, are natural plant polyphenolic compounds [20]. Proanthocyanidins have various biological effects, including antioxidant [21], anti-diabetic [22], and anti-ultraviolet activities [23]. Our previous research revealed that *C. camphora* leaves are abundant in proanthocyanidin-rich extracts (PCEs), which have strong antioxidant activity [24]. However, no research has concentrated on characterizing *C. camphora* PCEs and evaluating their anti-ultraviolet activity.

Traditional methods are generally employed for the separation of proanthocyanidins [25], which involve several disadvantages, which include involving toxic organic solvents, incurring low extraction efficiency, and being time consuming [25]. Ultrasonic-assisted extraction (UAE) has emerged as a novel method in separation science in these years. This is because UAE possesses the merits of cavitation effects, mechanical vibrations, and thermal effects under ultrasonic irradiation, which could rupture the plant cytoderm, facilitate the target compounds’ diffusion into solvents, and promote the dissolution of target compounds [26].

In this study, UAE was applied to extract PCEs from *C. camphora* leaves, and the extraction process was optimized by the Box–Behnken design (BBD). The phloroglucinolysis products of the PCEs were preliminarily analyzed using ultra-performance liquid chromatography–tandem mass spectrometry (UPLC–MS/MS). Furthermore, the anti-ultraviolet activity of the obtained *C. camphora* PCEs was studied to facilitate their integrated utilization and offer scientific data for their potential application as a natural sunscreen additive.

## 2. Results and Discussion

### 2.1. Optimization of the UAE Process Using the BBD

#### 2.1.1. Model Fit and Regression Coefficients

The UAE process for extracting *C. camphora* leaf PCEs was further optimized using BBD. A total of 29 runs associated with four variables were performed, and the results are given in Table 1. A quadric equation was acquired to analyze and validate the experimental results, as follows:

*Y* = 69.60 + 3.93*A* + 1.68*B* + 1.18*C* + 4.13*D* + 1.98*AB* + 1.65*AC* + 1.73*AD* + 1.49*BD* − 4.82*A*^2^ − 2.87*B*^2^ − 0.92*C*^2^ − 2.31*D*^2^ (1)

Table 2 details the results of the analysis of variance (ANOVA) on the PCE yield extracted by UAE. The *R*^2^ value was close to 1, which indicates that the mode fitted well to the response values [27]. Thus, the coefficient of determination (*R*^2^) was 0.9685, which demonstrates a strong correlation between the factors and PCE yield. The adjusted *R*^2^ (0.9370) logically coincides with the predicted *R*^2^ (0.8289), which shows that the model terms were significant [28]. The lack-of-fit’s *p* value (0.0740) and the model’s *F* value (29.72) suggest that the developed quadratic model was adequate for UAE process optimizing. Additionally, the low coefficient of variation value (*C*.*V*.% = 1.93) reflects that the developed model exhibited high credibility and good adaptability. The results of the ANOVA show that the independent terms of *A*, *B*, and *D*, the interactive term of *AD*, and the quadratic variables of *A*^2^, *B*^2^, and *D*^2^ were extremely significant; the interactive variable of *AB* and the independent variable of *C* were highly significant; and the interactive variables of *AC* and *BD* were significant.

#### 2.1.2. Response Contour Plot

**Figure 1:**
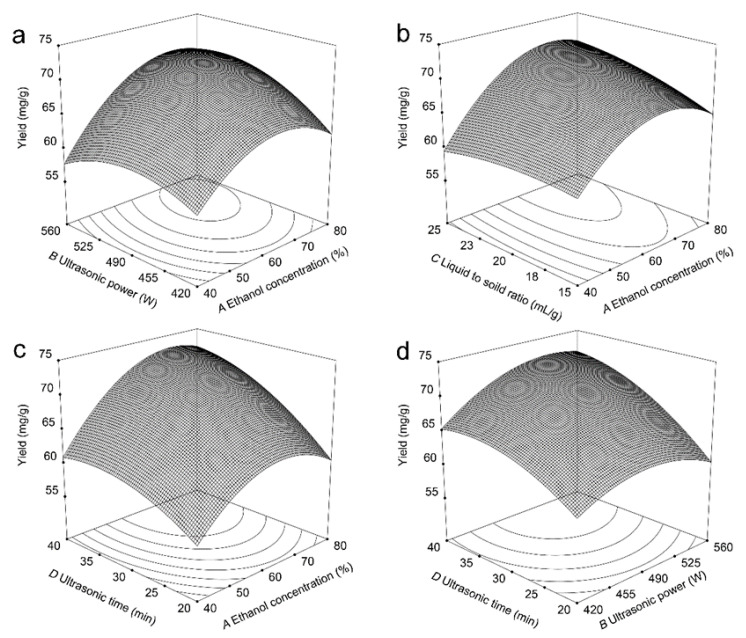
Three-dimensional surfaces generated from BBD: (**a**) the interaction effects of ethanol concentration (*A*) and ultrasonic power (*B*) on PCE yield; (**b**) the interaction effects of ethanol concentration (*A*) and liquid-to-solid ratio (*C*) on PCE yield; (**c**) the interaction effects of ethanol concentration (*A*) and ultrasonic time (*D*) on PCE yield; and (**d**) the interaction effects of ultrasonic power (*B*) and ultrasonic time (*D*) on PCE yield.

Figure 1 details the three-dimensional model of the response surface curve. As shown in Figure 1a, the interaction influences of ethanol concentration (*A*) and ultrasonic power (*B*) on the *C. camphora* leaf PCE yield were investigated. The *C. camphora* leaf PCE yield improved gradually with the enhancement of the ethanol concentration and ultrasonic power. Nevertheless, the increases of the ethanol concentration and ultrasonic power did not continuously enhance but decreased the PCE yield, which is consistent with the previous literature [29]. This may be because an increase in ethanol concentration raised the relative polarity of the solvent, which facilitated the expansion of plant cell walls and enlarged the contact area of the solute–solvent [29]. Meanwhile, a high ethanol concentration caused an increased polarity of the extraction solvent, which is likely to be inconvenient when extracting proanthocyanidins [30]. Figure 1b displays the interaction effects of ethanol concentration (*A*) and the liquid-to-solid ratio (*C*) on the PCE yield. The PCE yield improved with an enhancement in ethanol concentration and the liquid-to-solid ratio. This phenomenon may be caused by an appropriate improvement in the liquid-to-solid ratio, which can promote the circulation of substances, energy flow, and the penetration of the solvent into the solute [31,32,33]. In contrast, a very high liquid-to-solid ratio and excessive energy absorption in the extractant system could hinder extraction [33]. Figure 1c depicts the interaction effects of ethanol concentration (*A*) and ultrasonic time (*D*) on the PCE yield. The *C. camphora* leaf PCE yield tended to improve when the ethanol concentration and ultrasonic time were increased. This is likely due to the fact that increasing the ultrasonic time enlarges the contact area of solvents with solutes [34,35]. However, an additional increase in ultrasonic time incurred a decrease in the PCE yield. This may have been because a long ultrasonic time facilitated the frequent asymmetric collapse of microbubbles and a degradation of the target compounds [35,36]. Figure 1d illustrates the interaction effects of ultrasonic power (*B*) and ultrasonic time (*D*) on the PCE yield. The *C. camphora* leaf PCE yield first enhanced with an increase in ultrasonic power and time and then declined with an additional increase in these two variables. This may be due to the liquid circulation and tumult effects generated by ultrasonic cavitation, which can promote the movement of the solvent and enlarge the contact chance of the target compounds and the solvent, thus improving the extraction yield [37].

#### 2.1.3. Investigation of Model Adequacy

**Figure 2:**
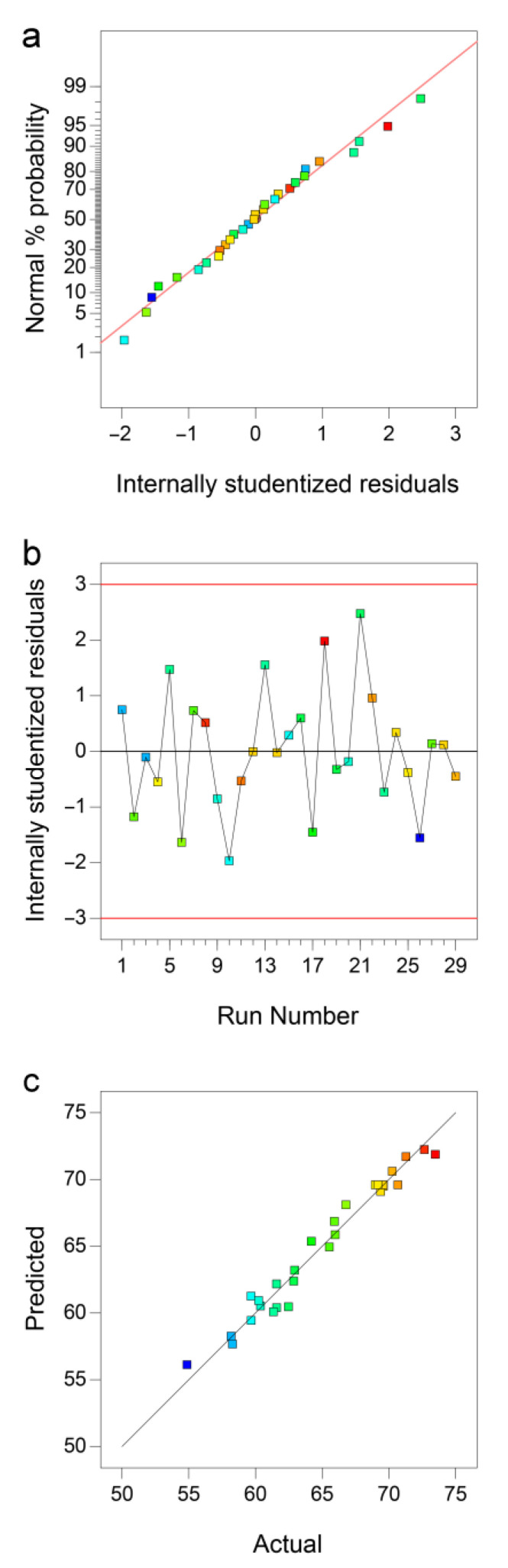
Three diagnostic charts for checking model adequacy. Normal plot of the residuals (**a**), the internally studentized residuals versus the run number (**b**), and a plot of the actual responses versus the predicted responses (**c**).

An investigation of the adequacy of the model could reveal whether the model produces incorrect or deceptive results. Figure 2 shows the three diagnostic charts for the adequacy test of the developed model, which include a normal graph, a graph of the residual versus run number, and a graph of the predicted versus actual responses. As shown in the normal graph (Figure 2a), the residuals of the response values normally spread as they lie closely on a straight line and do not reveal variance deviation. Figure 2b shows a plot of the residuals versus the run number. The good fit of the developed model was examined by establishing the internal studentized residuals versus the number of experimental runs, and it was found that all values fell randomly in the range of −3 to 3. A validation of the model is crucial, and this is accomplished by running several diagnostic tests. The predicted-versus-actual graph (Figure 2c) shows that the data points along with the straight line were linearly distributed, which suggests that the model was almost able to estimate the original experimental points [38]. According to these three diagnostic graphs, the established model is capable of the optimizing the UAE process for obtaining *C. camphora* leaf PCEs.

#### 2.1.4. Method Verification

The optimum UAE conditions acquired, on a laboratory scale, with a predicted *C. camphora* leaf PCE yield of 75.69 mg/g were as follows: 75% ethanol concentration, 24 mL/g of a liquid-to-solid ratio, an ultrasonic time of 39 min, and an ultrasonic power of 535 W. In consideration of the practical operability, the obtained conditions were slightly adjusted (an ultrasonic power of 540 W). Three validation experiments were performed to verify the precision and acceptability of the UAE process. The actual average yield of the *C. camphora* leaf PCEs was 77.46 ± 2.07 mg/g, which revealed that the conditions obtained by BBD were dependable.

### 2.2. Comparing UAE with Reference Methods

The extraction yields of the *C. camphora* leaf PCEs were 77.46 ± 2.07 mg/g, 68.56 ± 1.74 mg/g, and 55.15 ± 1.81 mg/g for UAE, HRE, and SE, respectively. On the basis of these results, UAE was obviously more efficient with the merits of environmental friendliness, short time consumption, high efficiency, and no destruction of active ingredients [39]. This is probably due to the mechanical effect, cavitation effect, and thermal effect produced by ultrasonic cavitation. Ultrasonic waves can spread through a series of compressional and evacuation waves through different molecules. At a sufficiently high ultrasonic power, liquid molecules are attracted to each other, thus creating cavitation bubbles. These bubbles grow gradually through a process of rectification and diffusion. In the process of separating natural products from plants, the suspended particles under the ultrasonication treatment can promote an asymmetric collapse of the bubbles, which results in the formation of microjets on the particles, thus disrupting their structure, increasing the mass transfer rate, and promoting a more efficient extraction [40,41].

### 2.3. Identifications of Phloroglucinolysis Products

**Figure 3:**
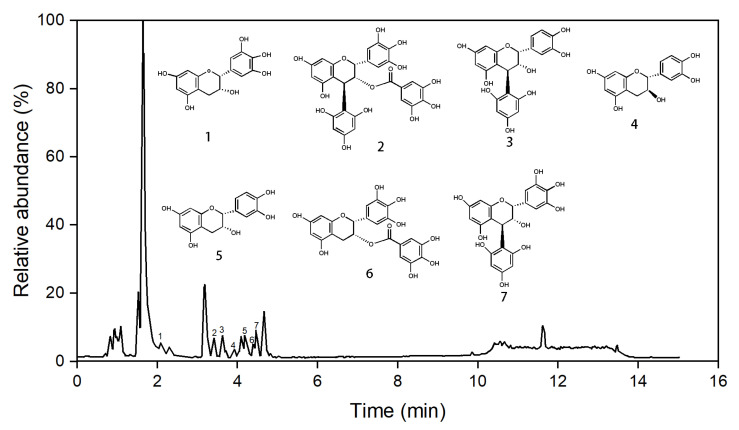
UPLC chromatograms of *C. camphora* leaf PCE of phloroglucinolysis samples at 280 nm, and the proposed structures of the phloroglucinolysis reaction products (peaks 1–7).

**Figure 4:**
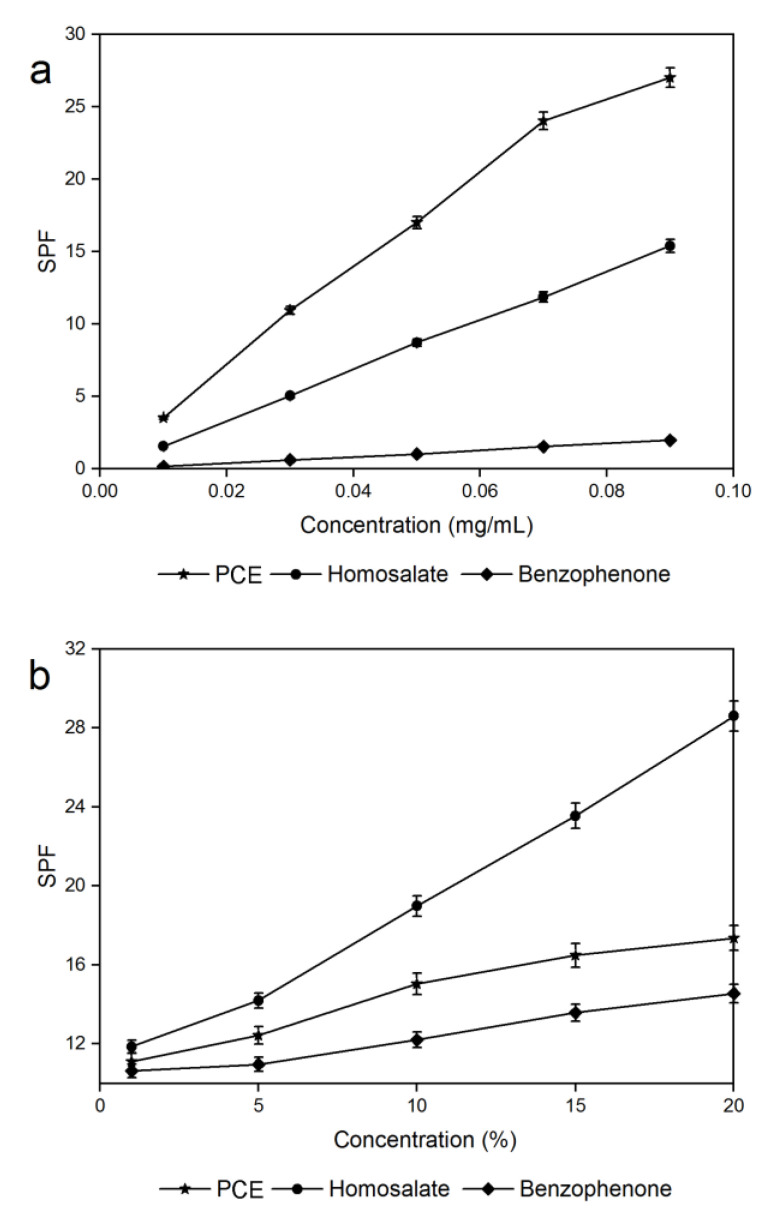
UVB protection capacities of *C. camphora* PCE ethanol solution (**a**) and sunscreen (**b**).

Figure 3 shows the UPLC chromatograms of the *C. camphora* leaf PCEs. The high-resolution mass was computed at negative mode to further characterize these peaks. The results are summarized in Figure 3, the Supplementary Materials, and Table 3. The identification of phloroglucinolysis products was on the basis of standards and a comparison with the literature. Manual characterization was performed by the investigating of the retention times, UV–vis spectra, and [M-H]^−^. Seven compounds with different molecular masses were identified in the tested samples. As shown in Figure 3 and Table 3, the retention times of these seven peaks exhibited on the chromatograms (Figure 3) were as follows: (1) 2.08 min, (2) 3.40 min, (3) 3.63 min, (4) 3.92 min, (5) 4.17 min, (6) 4.39 min, and (7) 4.48 min, respectively. On the basis of standards, four peaks (Table 3 and the Supplementary Materials) of 1, 4, 5, and 6 had their ion peaks at m/z 305.0669, 289.0719, 289.0716, and 455.0984, which were the intense [M–H]^−^ peaks of EGC, C, EC, and EGCG, respectively. Meanwhile, peaks 2, 3, and 7 gave the parent ion peaks ([M−H]^−^) at m/z 583.1103, 413.0876, and 430.0387, respectively. These peaks have been characterized by previous reports, and the present results are in accordance with reference data [42,43,44,45]. Therefore, the structure of these three peaks were analyzed as presented in Figure 4 and were identified as (−)-epigallocatechin-3-*O*-gallate-(4β-2)-phloroglucinol (EGCGP), epicatechin-(4β-2)-phloroglucinol (ECP), and epigallocatechin-(4β-2)-phloroglucinol (EGCP), respectively. Based on the literature [42,43], EGC, C, EC, and EGCG were recognized as terminal units and EGCG, EC, and EGC were identified as the extension units of *C. camphora* leaf PCE.

### 2.4. UVB Protection Efficacies

Figure 4 shows the results of the UVB protection capacity of the *C. camphora* PCEs. The sun protection factor (*SPF*) of the PCE ethanol solution gradually increased with an increasing concentration (Figure 4a). Meanwhile, the PCE ethanol solution showed higher *SPF* values than the commercially available sunscreen additives (benzophenone and homosalate) under the same concentration. This indicates that PCE ethanol solution has an excellent UVB-absorption ability. Figure 4b displays the *SPF* values of these three sunscreens under different concentrations. The *SPF* values of these three sunscreens increased when their concentrations increased. Compared to the commercially available sunscreens, the PCE sunscreen was inferior to homosalate but superior to benzophenone, indicating that *C. camphora* PCEs are a promising alternative to the commercial additives used as a natural sunscreen additive. Based on the study by Sierra-Cruz et al., proanthocyanidins are a class of flavanol monomers and polyphenolic compounds of their polymers [46]. The present study also shows that *C. camphora* PCEs are made from polymerized EGC, C, EC, and ECG. The common structural features of these units are aromatic rings and phenolic hydroxyl groups, so we deduced three reasons as to why the *C. camphora* PCEs had a desirable UVB-protection ability. (1) The aromatic rings and the phenolic structure exhibit an important role in UV absorption [47,48]. (2) Proanthocyanidins can scavenge reactive oxygen species generated by UV [49,50]. (3) Proanthocyanidins can regulate several signaling pathways in vivo to repair UVB-induced DNA damage [23,50]. This is consistent with the results of previous studies, in which proanthocyanidins possessed good UV absorption [51,52].

## 3. Materials and Methods

### 3.1. Materials and Reagents

*C. camphora* leaves were collected from the Jiangxi Normal University schoolyard (Nanchang, China) and were identified by Professor Ronggen Deng (Jiangxi Normal University, Nanchang, China). Fresh leaves were placed in the shade for 7 days and were then powdered and sieved. The powdered samples were defatted by soaking in 2 L of petroleum ether (boiling range, 60–90 °C) for 4 h, and a rotary evaporator was used to recover petroleum ether from the supernatant. The defatted leaf powders were dried before further treatment.

A pure cream (NIVEA refreshingly soft moisturizing cream) was applied to prepare sunscreen. Four standards of epigallocatechin (EGC), AB-8 macroporous resins, catechin (C), homosalate, epicatechin (EC), benzophenone, and (−)-epigallocatechin-3-*O*-gallate (EGCG) were purchased from Aladdin Reagent (Shanghai, China). Other chemicals and reagents were purchased from Yuanye Biotechnology Co., Ltd. (Shanghai, China) and were used without further treatment.

### 3.2. Extraction of C. camphora Leaf PCEs by UAE

For UAE, the de-oiled *C. camphora* leaf powder (5 g), coupled with an extraction solvent, was put into a glass flask and placed in an ordinary ultrasonic cleaning bath for extracting PCEs. After extraction, the suspension mixture was filtered, and then the supernatant liquid was evaporated to recover the extraction solution. The acidic butanol method was used to determine the PCE content, as described by Han et al. [53]. A standard curve for the determination of PCEs was obtained as *Y* = 3.5425*X* + 0.0026 (*R*^2^ = 0.9999) with a good linearity in the scope of 0.0625 to 1.00 mg/mL. The PCE yield was conveyed as milligrams of PCE equivalent per gram of leaf powder. Before the further structural identification and evaluation of in vitro anti-ultraviolet activity, AB-8 macroporous resins were used to purify the crude *C. camphora* leaf extracts, as described by Liu et al. [54].

### 3.3. Experimental Design and Optimization

Four factors were further investigated during UAE, which include the ethanol concentration (*A*), ultrasonic power (*B*), liquid-to-solid ratio (*C*), and ultrasonic time (*D*). The appropriate influence ranges of all variables for BBD optimization were determined through pre-test experiments. A quadratic polynomial Formula (2) was employed to fit the UAE process, as follows:

(2) $$Y=\beta_{0}+\sum_{i=1}^{k}\beta_{i}X_{i}+\sum_{i=1}^{k}\beta_{ii}X_{i}^{2}+\sum_{i=1}^{k}\sum_{j=i+1}^{k}\beta_{ij}X_{i}X_{j}$$

where *Y* (mg/g) denotes the estimated yield; *β*_0_, *β_i_*, *β_ii_*, and *β_ij_* denote the intercept, linear, quadratic, and interactive coefficients, respectively; *k* denotes the times of the tested factors; and *X_i_* and *X_j_* denote the independent factors.

### 3.4. Comparing UAE with Reference Extraction Methods

Heat reflux extraction (HRE) and soaking extraction (SE) were used as reference methods to compare with UAE for extracting *C. camphora* PCEs. HRE was performed in an electric jacket at 1 kW for 4 h, and the other factors were kept consistent with the obtained optimal conditions of UAE. SE was carried out by soaking for 24 h at a 7 mL/g liquid-to-solid ratio. The extracted suspension was filtered, and the extraction solution was evaporated to recover the solvent before determining the content of the PCEs.

### 3.5. Identification of PCE Phloroglucinolysis Products by UPLC–MS/MS Analysis

**Figure 5:**
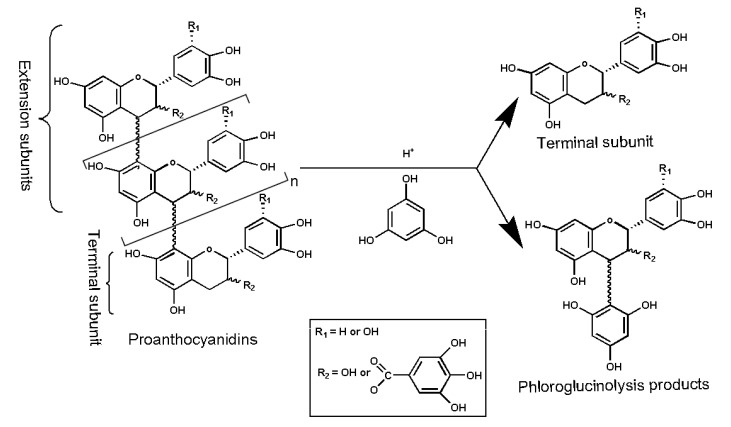
Reaction pathway of phloroglucinolysis for proanthocyanidins.

The *C. camphora* leaf PCEs were hydrolyzed in the presence of phloroglucinol using acid catalysis, as previous studies have reported [55,56], and the reaction pathway of phloroglucinolysis for proanthocyanidins is presented in Figure 5. An HCl acidic methanol solution (0.1 N), 50 mg of the PCE samples, 800 μL of phloroglucinol (50 mg/mL), and 10 mg/mL of vitamin C were mixed in a test tube with a stopper and, finally, were incubated for 20 min at 50 °C. After incubation, 5 volumes of 40 mmol of sodium acetate were added to the tube and were cooled by an ice bath. The obtained mixture was eventually filtrated by 0.45 μm nylon membranes before further analysis.

UPLC–MS/MS was performed using a Thermo Scientific Vanquish instrument (Thermo, Dreieich, Germany). The process used an Acquity UPLC^®^ BEH C18 (2.1 mm × 100 mm, 1.7µm, Thermo, Dreieich, Germany) to separate the compounds. The elution solvent was formic acid with 1% volume fraction, dissolved in different ratios of acetonitrile (*A*) and water (*B*), and the following chromatographic conditions were used: 0 min (*A* 5%: *B* 95%), 1 min (*A* 5%: *B* 95%), 3 min (*A* 30%: *B* 70%), 7 min (*A* 60%: *B* 40%), 9 min (*A* 95%: *B* 5%), 12 min (*A* 95%: *B* 5%), 12.1 min (*A* 5%: *B* 95%), and 15 min (*A* 5%: *B* 95%). The injection volume of the samples was 1 μL. During the detection process, the column temperature was held at 25 °C with a flow rate of 0.25 mL/min. The mass spectrometry procedure was carried out on a Q Exactive quadrupole/electrostatic field orbit trap high-resolution mass spectrometer (Thermo, Dreieich, Germany). The mass spectrometry was operated in positive ion mode with a 3.7 kV of spray voltage, and the full scan mass ranged from 100 to 1000 m/z. The following optimal source parameters were used: capillary voltage, 3.0 kV; cone voltage, 10 V; cone gas flow, 80 L/h; auxiliary gas-heater temperature 350 °C; capillary temperature, 320 °C, desolvation gas, nitrogen; and flow rate, 600 L/h. The analytical standards of C (98%, HPLC grade) and EC (98%, HPLC grade) were applied for a confirmation of the suitability of the UPLC–MS/MS method for the identification of PCE phloroglucinolysis products.

### 3.6. Determination the UVB-Protection Performance of C. camphora PCEs

In order to confirm the anti-ultraviolet activity of *C. camphora* PCEs, a preliminary study on the evaluation of the anti-ultraviolet activity of the PCE ethanol solution was carried out. After this, practical application tests were conducted to determine the UVB-protection ability of PCE sunscreen. The UVB-protection performance of the *C. camphora* PCE ethanol solution was determined by the UV spectrophotometric method. The original PCE master batch was diluted with ethanol to obtain the concentrations of 10, 30, 50, 70, 90, and 100 μg/mL and was then measured. Each sample was tested three times at 5 nm intervals from 290 to 320 nm with a 1 cm quartz cuvette, and ethanol was used as a blank control.

The preparation process of the *C. camphora* PCE sunscreen referred to a previous study [57]. The *C. camphora* PCEs were mixed with the pure cream to reach the final ratios of 1 wt%, 5 wt%, 10 wt%, 15 wt%, and 20 wt%, respectively. Homosalate and benzophenone were used as positive controls. The prepared sunscreens were evenly applied to Basewing medical tape (2 mg per cm^2^), were taped on the clean side of the quartz cuvette, and were then dried in the dark for 20 min. The UVB absorbance of each specimen was measured by a UV spectrometer. The absorbance was determined three times at 5 nm intervals in the wavelength range of 290–320 nm.

Standardized testing of sunscreen products for the evaluation of *SPF* can provide consistent product efficacy values for consumers worldwide [58]. For this purpose, the *SPF* values of the *C. camphora* PCE ethanol solutions and sunscreen were calculated to evaluate their sun protection ability. The *SPF* values were measured as described by Mansur et al. [59], as follows:

(3) $$SPF=CF\times \sum_{290}^{320}\left[E(\lambda )\times I(\lambda )\times Abs(\lambda )\right]$$

where *E*(λ) signifies the erythemal effect spectrum, *Ι*(λ) denotes sunlight intensity, *Abs*(λ) means absorbance intensity, and *CF* represents the correction factor (=10). The value of *E*(λ) multiplied by *I*(λ) is based on Sayre et al. [60].

### 3.7. Statistical Analysis

BBD was conducted through the Design Expert 8.0 program (Stat-Ease, Minneapolis, MN, USA). An ANOVA test was conducted to investigate the significance of variation in the *C. camphora* leaf PCE yield. Each experiment was repeated three times (*n* = 3). Based on pre-installed defaults, the actual PCE yield for each test was expressed as an average, and other data were conveyed as averages ± standard deviations.

## 4. Conclusions

UAE was employed to separate *C. camphora* leaf PCEs, and the extraction process was optimized by BBD. The optimal conditions of UAE, on a laboratory scale, with a real PCE yield of 77.46 ± 2.07 mg/g were as follows: a 75% ethanol concentration, a 24 mL/g of liquid-to-solid ratio, an ultrasonic time of 39 min, and an ultrasonic power of 540 W. UAE was more efficient for separating PCEs compared with traditional extraction methods. The extension and terminal units of the *C. camphora* leaf PCEs were preliminarily identified by analyzing their phloroglucinolysis products with the UPLC–MS/MS method. Furthermore, the *C. camphora* leaf PCEs showed strong UV protection compared with commercial additives, which are expected to be developed as a natural sunscreen additive in the cosmetics industry.

## Figures and Tables

**Table 1:** Box–Behnken design matrix, and the actual and predicted values for the yields of *C. camphora* PCEs.

Run	*A*	*B*	*C*	*D*	Yield (mg/g)	
					Actual	Predicted
1	40 (−1)	560 (0)	20 (0)	30 (0)	58.28	57.67
2	60 (0)	560 (0)	15 (−1)	30 (0)	65.91	66.86
3	40 (−1)	420 (−1)	20 (0)	30 (0)	58.18	58.26
4	60 (0)	490 (0)	20 (0)	30 (0)	68.98	69.60
5	40 (−1)	490 (0)	15 (−1)	30 (0)	61.59	60.40
6	60 (0)	560 (0)	25 (+1)	30 (0)	66.78	68.10
7	80 (+1)	490 (0)	15 (−1)	30 (0)	65.54	64.95
8	80 (+1)	490 (0)	20 (0)	40 (+1)	72.66	72.24
9	40 (−1)	490 (0)	20 (0)	40 (+1)	60.24	60.93
10	60 (0)	490 (0)	15 (−1)	20 (−1)	59.67	61.26
11	60 (0)	560 (0)	20 (0)	40 (+1)	71.28	71.71
12	60 (0)	490 (0)	20 (0)	30 (0)	69.59	69.60
13	60 (0)	420 (−1)	20 (0)	20 (−1)	61.35	60.09
14	60 (0)	490 (0)	20 (0)	30 (0)	69.57	69.60
15	40 (−1)	490 (0)	25 (+1)	30 (0)	59.68	59.45
16	60 (0)	420 (−1)	15 (−1)	30 (0)	62.87	62.39
17	60 (0)	420 (−1)	20 (0)	40 (+1)	64.19	65.37
18	60 (0)	490 (0)	25 (+1)	40 (+1)	73.48	71.88
19	60 (0)	490 (0)	25 (+1)	20 (−1)	62.94	63.20
20	80 (+1)	490 (0)	20 (0)	20 (−1)	60.38	60.53
21	60 (0)	560 (0)	20 (0)	20 (−1)	62.47	60.47
22	60 (0)	490 (0)	20 (0)	30 (0)	70.67	69.60
23	80 (+1)	420 (−1)	20 (0)	30 (0)	61.58	62.17
24	60 (0)	490 (0)	15 (−1)	40 (+1)	69.38	69.10
25	60 (0)	490 (0)	20 (0)	30 (0)	69.17	69.60
26	40 (−1)	490 (0)	20 (0)	20 (−1)	54.87	56.13
27	60 (0)	420 (−1)	25 (+1)	30 (0)	65.97	65.86
28	80 (+1)	560 (0)	20 (0)	30 (0)	69.58	69.48
29	80 (+1)	490 (0)	25 (+1)	30 (0)	70.25	70.61

*A*: ethanol concentration, %; *B*: ultrasonic power, W; *C*: liquid-to-solid ratio, mL/g; *D*: ultrasonic time, min.

**Table 2:** Analysis of variance (ANOVA) for the fitted quadratic model of *C. camphora* PCE extraction determined from the Box–Behnken design.

Source	Sum of Squares	Degree of Freedom			Mean Square	*F* Value	*p* Value
Model ^a^	675.67	14			48.26	30.75	<0.0001 ***
*A*	185.26	1			185.26	118.04	<0.0001 ***
*B*	33.87	1			33.87	21.58	0.0004 ***
*C*	16.66	1			16.66	10.62	0.0057 **
*D*	204.60	1			204.60	130.37	<0.0001 ***
*AB*	15.60	1			15.60	9.94	0.0070 **
*AC*	10.96	1			10.96	6.98	0.0193 *
*AD*	11.94	1			11.94	7.61	0.0154 *
*BC*	1.24	1			1.24	0.79	0.3885
*BD*	8.91	1			8.91	5.68	0.0319 *
*CD*	0.17	1			0.17	0.11	0.7454
*A* ^2^	150.96	1			150.96	96.19	<0.0001 ***
*B* ^2^	53.54	1			53.54	34.11	<0.0001 ***
*C* ^2^	5.50	1			5.50	3.50	0.0823 *
*D* ^2^	34.74	1			34.74	22.14	0.0003 ***
Residual	21.97	14			1.57		
Lack of fit	20.26	10			2.03	4.72	0.0740
Pure error	1.72	4			0.43		
Cor total ^b^	697.64	28					
Credibility analysis of the regression equations							
*Std. Dev.* ^c^	Mean	*C.V.* ^d^ %	Press	*R* ^2^	Adjust *R*^2^	Predicted *R*^2^	Adequacy precision
1.25	65.07	1.93	119.36	0.9685	0.9370	0.8289	17.8880

^a^ *A*: ethanol concentration, %; *B*: ultrasonic power, W; *C*: liquid-to-solid ratio, mL/g; *D*: ultrasonic time, min; ^b^ totals of all information corrected for the mean; ^c^ standard deviation; ^d^ coefficient of variation; * *p* < 0.1, significant; ** *p* < 0.01, highly significant; *** *p* < 0.001, extremely significant.

**Table 3:** Computed high-resolution mass (negative mode), molecular weight, and fragment ions of all compounds identified in *C. camphora* PCE phloroglucinolysis reaction products.

No.	Retention Time (min)	Parent Ion (m/z)	Molecular Formula	Compound Identified ^a^	Tentatively Identified
1	2.08	305.0669	C_15_H_14_O_7_	EGC	[42]
2	3.40	583.1103	C_28_H_22_O_14_	EGCGP	[43]
3	3.63	413.0876	C_21_H_18_O_9_	ECP	[43]
4	3.92	289.0719	C_15_H_14_O_6_	C	Standard
5	4.17	289.0716	C_15_H_14_O_6_	EC	Standard
6	4.39	455.0984	C_22_H_18_O_11_	EGCG	[44]
7	4.48	430.0387	C_21_H_18_O_10_	EGCP	[45]

^a^ EGC (epigallocatechin), EGCGP ((−)-epigallocatechin-3-*O*-gallate-(4β-2)-phloroglucinol), ECP (epicatechin-(4β-2)-phloroglucinol), C (catechin), EC (epicatechin), (−)-epigallocatechin-3-*O*-gallate (EGCG), and EGCP (epigallocatechin-(4β-2)-phloroglucinol).

## Data Availability

The data presented in this study are available on request from the corresponding author.

## Supplementary Materials

The following supporting information can be downloaded at: https://www.mdpi.com/article/10.3390/molecules29040796/s1. Figure S1: MS fragmentation pattern of cleavage products (peak 1), Figure S2: MS fragmentation pattern of cleavage products (peak 2), Figure S3: MS fragmentation pattern of cleavage products (peak 3), Figure S4: MS fragmentation pattern of cleavage products (peak 4), Figure S5: MS fragmentation pattern of cleavage products (peak 5), Figure S6: MS fragmentation pattern of cleavage products (peak 6), and Figure S7: MS fragmentation pattern of cleavage products (peak 7).
