# Supplementary material for: Characterization and Anti-Ultraviolet Radiation Activity of Proanthocyanidin-Rich Extracts from Cinnamomum camphora by Ultrasonic-Assisted Method
Source: Molecules. 2024 Feb 8;29(4):796. doi: 10.3390/molecules29040796 (PMC10893137; doi:10.3390/molecules29040796)
Supplement: Supplementary file 1 [file molecules-29-00796-s001.zip › molecules-2842152-supplementary.pdf]

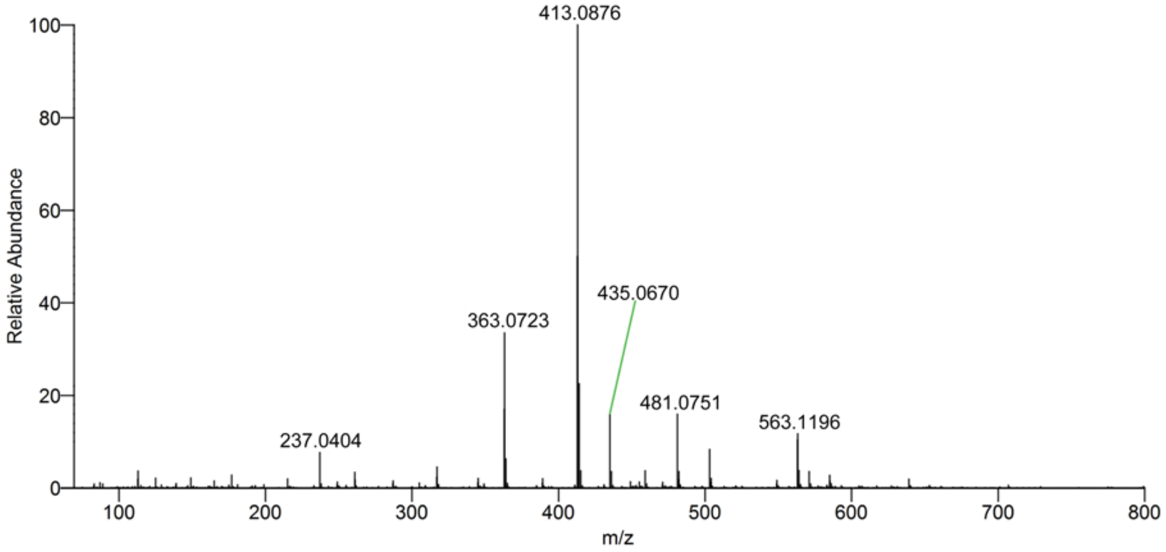

Figure S1 MS fragmentation pattern of cleavage products (peak 1)

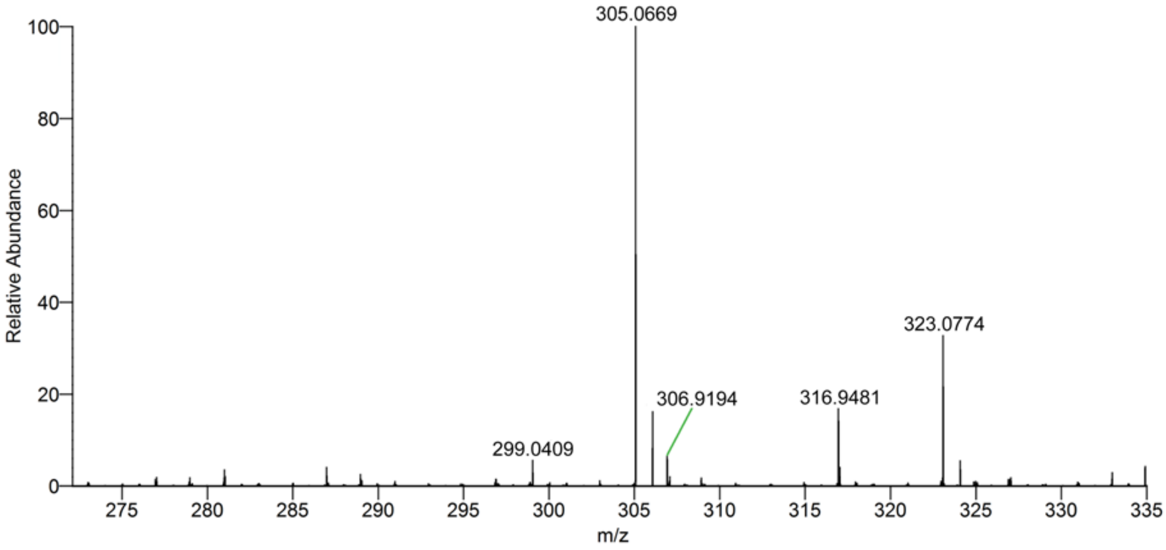

Figure S2 MS fragmentation pattern of cleavage products (peak 2)

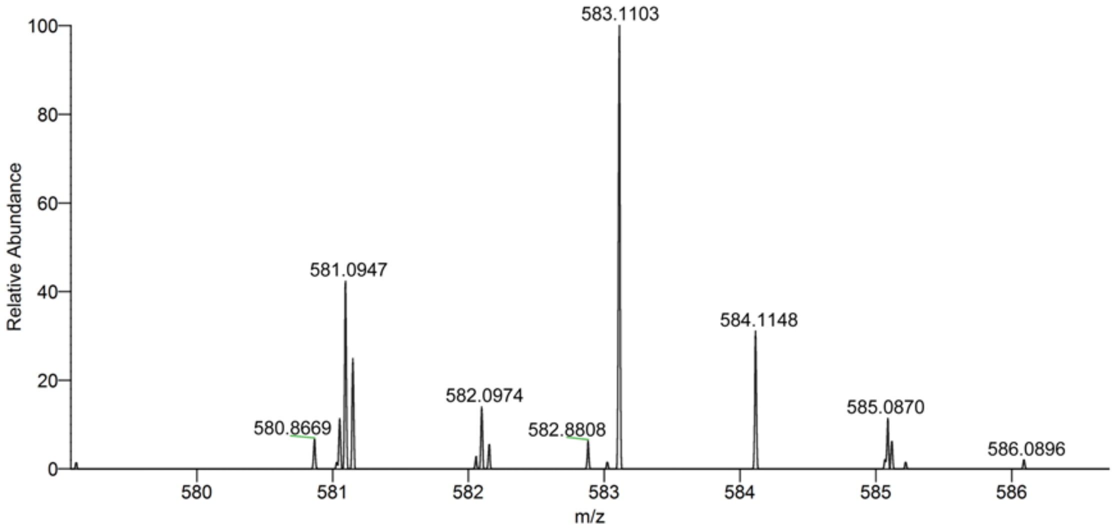

Figure S3 MS fragmentation pattern of cleavage products (peak 3)

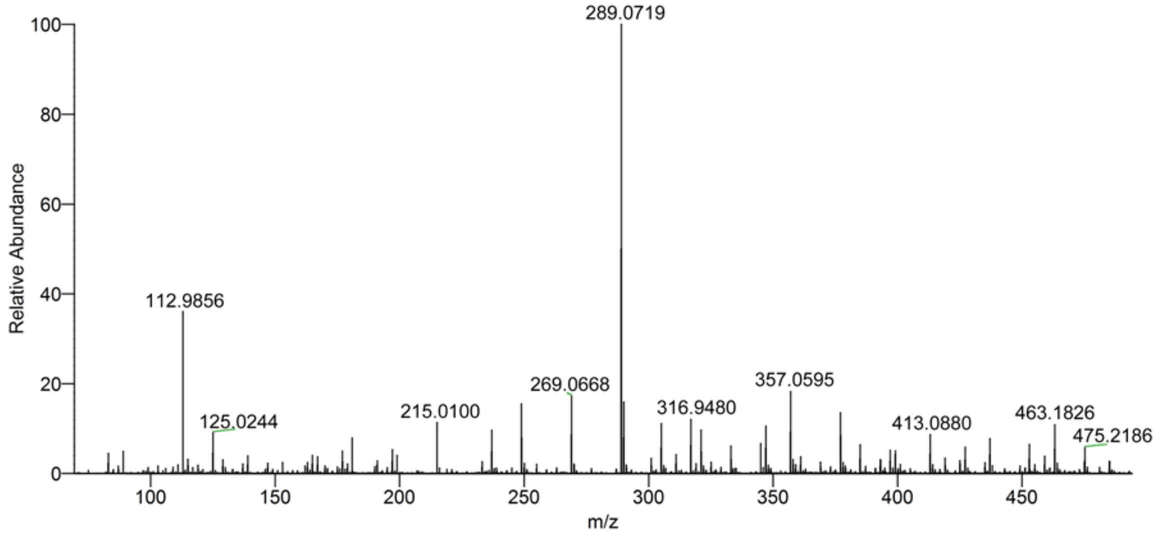

Figure S4 MS fragmentation pattern of cleavage products (peak 4)

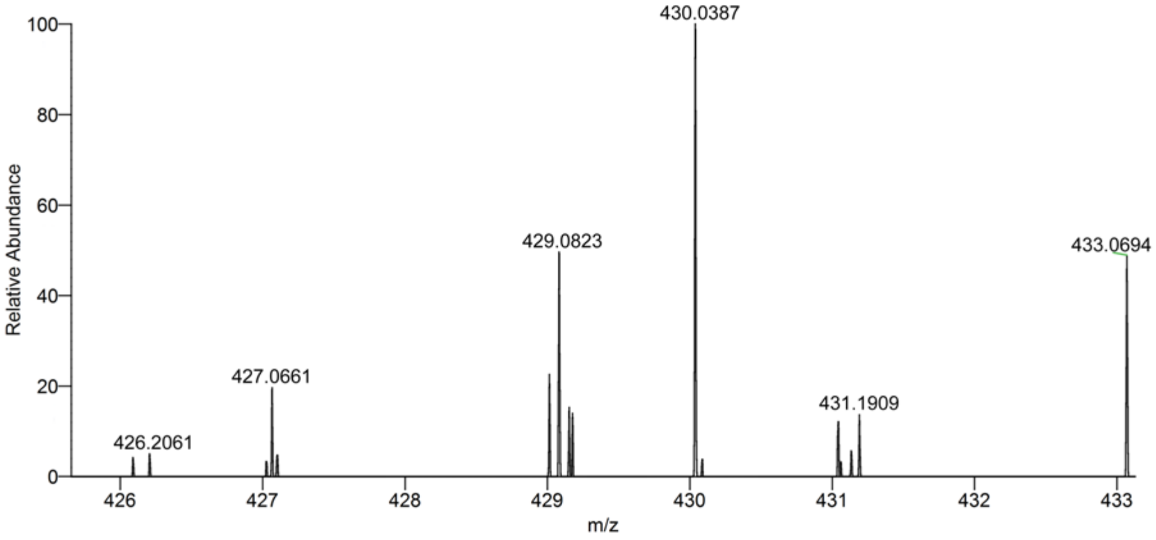

Figure S5 MS fragmentation pattern of cleavage products (peak 5)

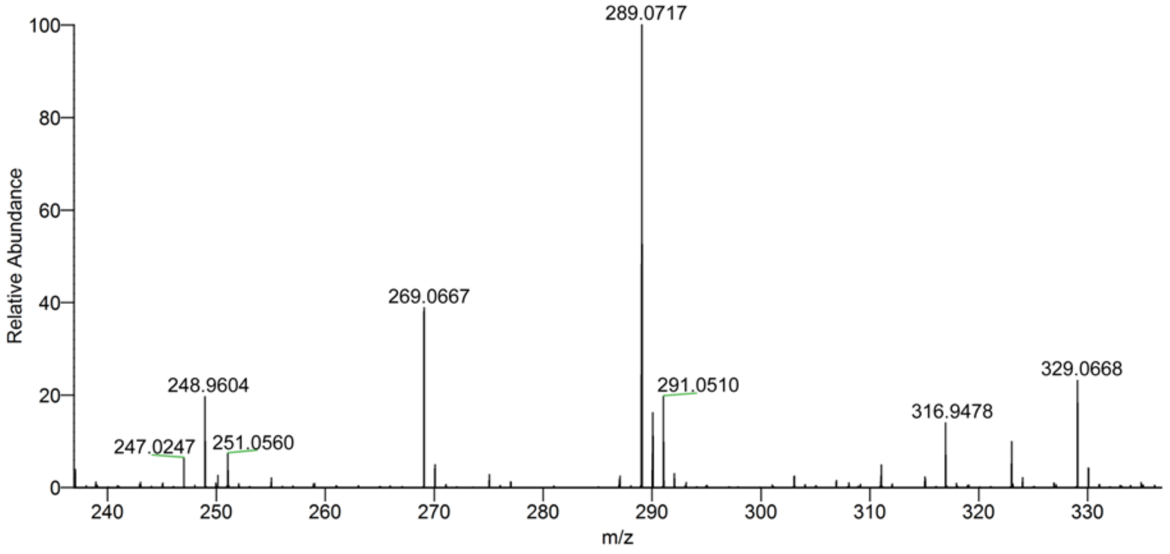

Figure S6 MS fragmentation pattern of cleavage products (peak 6)

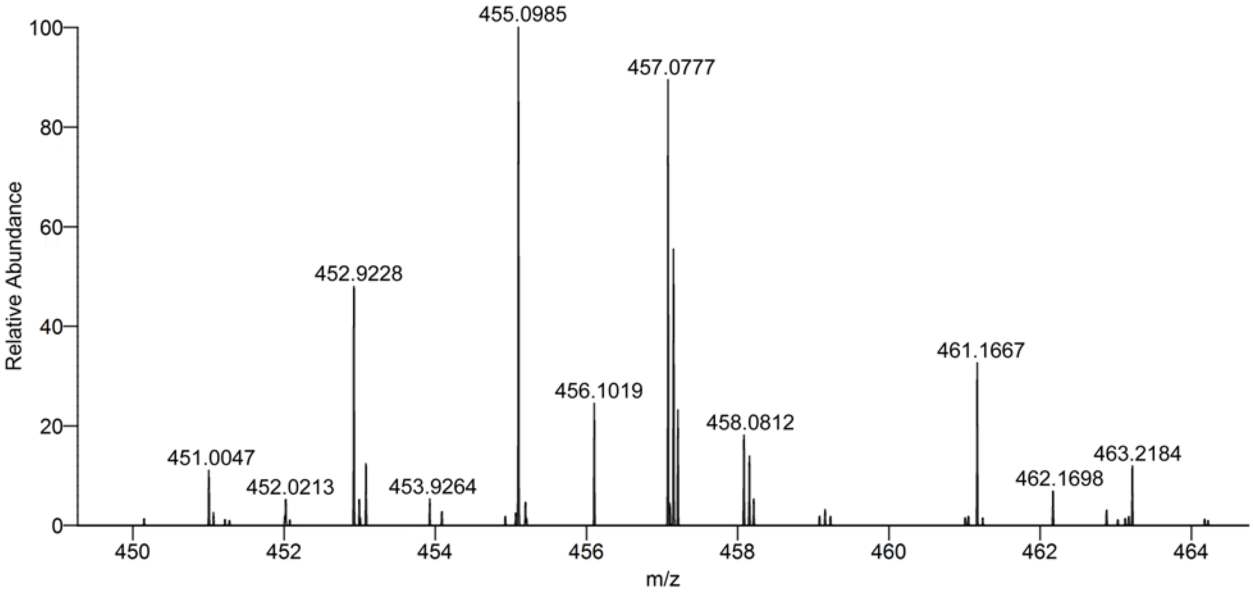

Figure S7 MS fragmentation pattern of cleavage products (peak 7)
